# Environmental impact of tsetse eradication in Senegal

**DOI:** 10.1038/s41598-019-56919-5

**Published:** 2019-12-30

**Authors:** Mamadou Ciss, Mireille D. Bassène, Momar T. Seck, Abdou G. Mbaye, Baba Sall, Assane G. Fall, Marc J. B. Vreysen, Jérémy Bouyer

**Affiliations:** 1 Institut Sénégalais de Recherches Agricoles, Laboratoire National d’Elevage et de Recherches Vétérinaires, BP 2057 Dakar, Hann Sénégal; 2 Ministère de l’Elevage et des Productions animales, Direction des Services Vétérinaires, BP 45677 Dakar, Sénégal; 3 Insect Pest Control Laboratory, Joint FAO/IAEA Programme of Nuclear Techniques in Food and Agriculture, A-1400 Vienna, Austria; 4 Unité Mixte de Recherche ‘Interactions hôtes-vecteurs-parasites-environnement dans les maladies tropicales négligées dues aux trypanosomatides’, Centre de Coopération Internationale en Recherche Agronomique pour le Développement (CIRAD), 34398 Montpellier, France; 5 Unité Mixte de Recherche ‘Animal, Santé, Territoires, Risques et Ecosystèmes’, Centre de Coopération Internationale en Recherche Agronomique pour le Développement (CIRAD), 34398 Montpellier, France

**Keywords:** Environmental biotechnology, Conservation biology

## Abstract

The sterile insect technique is an environment friendly control tactic and is very species specific. It is not a stand-alone technique and has been used mostly in combination with other control tactics within an area-wide integrated pest management strategy. For a period of eight years, the direct impact of a campaign to eradicate a population of the tsetse fly *Glossina palpalis gambiensis* in Senegal was monitored using a set of fruit-feeding insect species (Cetoniinae and Nymphalidae) that served as ecological indicators of the health of the ecosystem. Here we show that the eradication campaign had very limited impacts on the apparent densities of the most frequent species as well as three diversity indexes during the reduction phase involving insecticides but reverted to pre-intervention levels as soon as the release of the sterile male insects started. These results greatly expand our understanding of the impact of vector eradication campaigns on non-target species.

## Introduction

Tsetse are the vectors of human African trypanosomosis (HAT) or sleeping sickness^1^ and African animal trypanosomosis (AAT) or nagana^2^. In the sub-humid savannah of West Africa, riverine tsetse species such as *Glossina palpalis gambiensis* (Vanderplank 1949) inhabit riparian forests and is one of the major vectors of these diseases^3^. In Senegal, as in other parts of West Africa, AAT is a major obstacle to the development of more efficient and sustainable livestock production systems^4,5^. In 2005, the Government of Senegal initiated a project entitled “Projet de lutte contre les glossines dans les Niayes” (Tsetse control project in the Niayes) with the aim of creating a zone free of *G. p. gambiensis*. During a feasibility study^6^ the tsetse-infested area proved to be delimited to 1 000 km^2^ and the impact of animal trypanosomosis on the farmers’ welfare was quantified, with annual benefits of 2.8 million Euro should the tsetse fly population be removed^4^. An area-wide integrated pest management (AW-IPM) strategy^7^ was formulated that included a sterile insect technique (SIT) component^8^ to eradicate the isolated tsetse populations from the Niayes^9^. In view of the extreme fragmentation of the tsetse habitat in the Niayes and the high human population density (peri-urban area), the AW-IPM strategy excluded aerial spraying of insecticides^10^. The tsetse fly populations were suppressed using insecticide-impregnated traps/targets and “pour-on” for cattle including a mixture of repellents and insecticides^11^, followed by the release of sterile males to eliminate the remaining relic fly pockets^9^. Here we show that the strategy selected and implemented had minimal impact on non-target species.

There is few reliable literature available that documents the impact of tsetse control operations on the environment. It is obvious that early tsetse control operations such as extermination of game animals and destruction of favorable tsetse habitat were extremely detrimental to the environment^12^. Residual insecticides (mainly organochlorines)^13^ were commonly used in tsetse control operations in the 1950–1960’s but were gradually replaced thereafter by ultra-low-volume non-residual insecticide techniques that were less polluting^12^, i.e. the sequential aerosol technique (SAT) that mainly relied on the insecticides endosulfan and pyrethroids^10^. In the 1970–1980’s, bait technologies such as insecticide-impregnated targets and traps became more widely adopted and these were considered more friendly to the environment in view of their specificity for tsetse flies, with the exception of some dipteran biting flies such as Tabanidae and Stomoxyinae. However, their deployment, maintenance and frequent sampling checks requires the clearing of vegetation for access tracks and for increasing their visibility in the trapping site, and this has negative effects on the environment that have largely been ignored. In addition, in protected areas, these tracks might provide easy access for illegal hunters and poachers^12^, and their deployment can affect habitat use by birds and large mammals, i.e. the presence of targets reduced the frequency of occurrence of antelopes, suids and large herbivores, but small carnivores, monkeys, rodents and hares remained unaffected. Moreover, in the areas where targets were deployed, more than half of the recorded species had lower frequencies indices than in the sites without targets^14^.

Another study assessed the impact of insecticide pour-ons^15^ on dung beetles^16,17^. Pour-ons are oily insecticide formulations that are poured directly on livestock and as such can contaminate the dung mainly through licking and from the peri-anal area. As a result, dung fauna populations can significantly be depleted and in view of their crucial role of burying fresh dung, negatively affect soil structure, water holding capacity and soil fertility.

In 2001 and 2002, a population of *Glossina morsitans centralis* was eradicated from the Okavango Delta in Botswana^18^ using the SAT. A study that assessed the impact of the SAT on non-target invertebrates indicated that population density of both aquatic and terrestrial invertebrates were reduced immediately after the sprays but most populations eventually recovered to pre-spray numbers^19^.

No impact on non-target species is expected from the SIT as this control tactic is species-specific and does not entail the release of any harmful product in the environment^20^.

## Results

**Figure 1:**
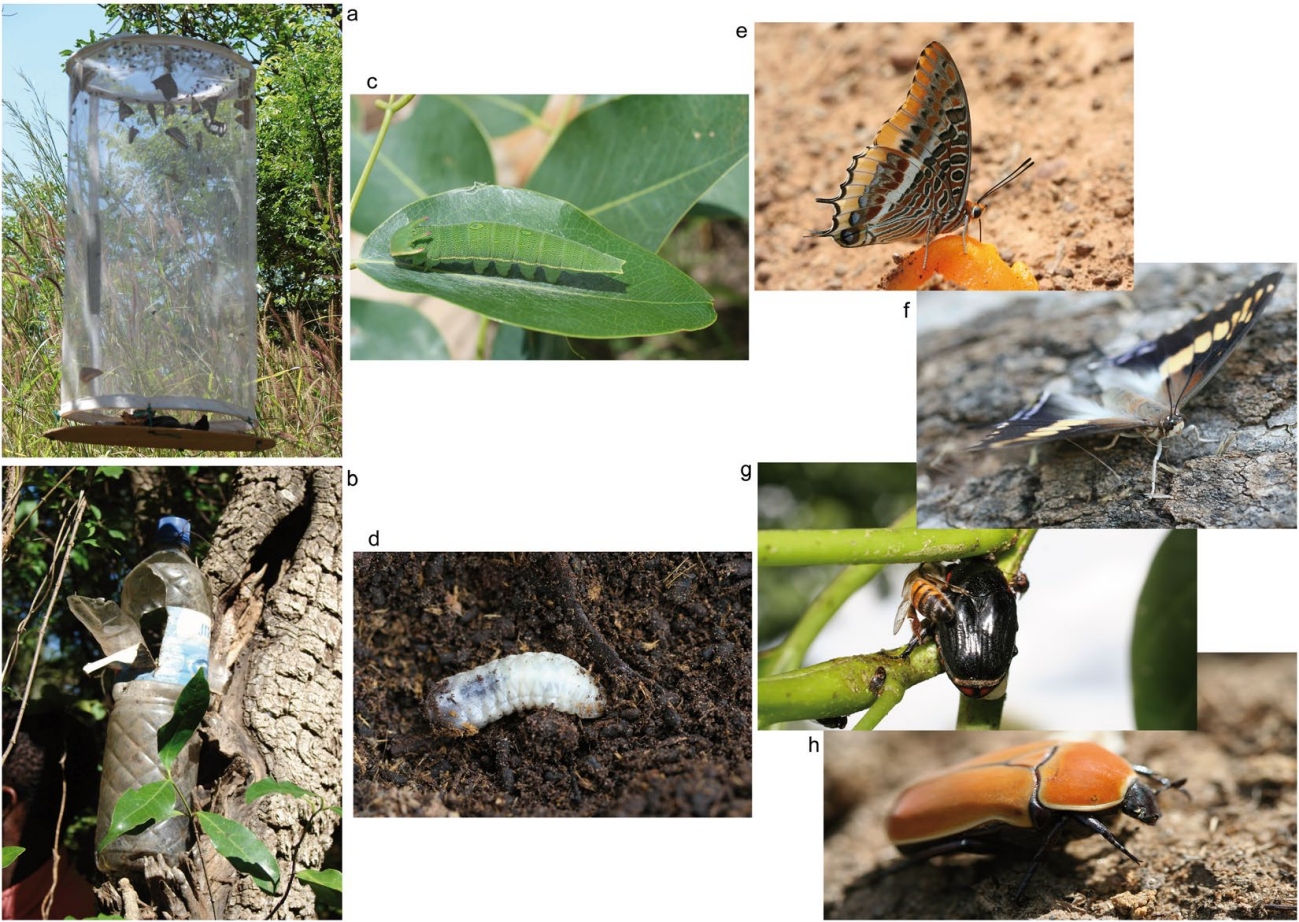
Illustration of traps and some ecological indicators used for the environmental monitoring in Senegal: banana trap for fruit feeding butterflies (**a**), banana trap for fruit feeding beetles (**b**), larval stages of *Charaxes sp.* (**c**) that feed on trees and of Cetoniinae (**d**) that are detritivorous and live in plant litter. Adult *Charaxes epijasius* feeding on a mango (**e**), *Charaxes achemenes* resting on a trunk (**f**), *Chondrorrhina abbreviata* feeding on a plant wound together with a bee (**g**) and *Pachnoda marginata* on the ground (**h**).

In this study, the environmental impact of a tsetse eradication program in the Niayes of Senegal was assessed using an innovative monitoring strategy based on fruit-feeding insect species that served as ecological indicators. These were previously described as sensitive to fluctuations in the health of savannah ecosystems in Burkina Faso and Benin^21^. Whereas these ecosystems are very similar to those in Senegal, such a strategy has never been applied in a tsetse eradication program before. Two insect families were selected as ecological indicators (Coleoptera: Scarabaeidae (Cetoniinae) and Lepidoptera: Nymphalidae) and they have the advantage that the adults can be easily trapped in standard banana-baited traps (Fig. 1). The ecological roles and trophic levels of their larval stages are however completely different (saprophytic versus phytopagous respectively) and their presence and density provide an optimal indication of the health of the global ecosystem (see details in the method section). Their characteristics include taxonomical and ecological diversity, ecological fidelity, sedentary (species with short dispersal range), endemicity (non-invasive species), taxonomical knowledge and ease of identification, abundance and ease of monitoring, indication of other species and specific resources, temporal and spatial fluctuations and predictable, rapid, sensitive, and a linear response to disturbance that is easy to analyze^21,22^. The seasonal variation of the apparent densities of these ecological indicators is the only limitation and therefore, the monitoring must be done during the same period of the year^23^. For this study, the traps were deployed for eight consecutive years in October-November, a period that coincides with the end of the rainy season – beginning of the cold dry season and is the period of their highest diversity and density. In addition, the tsetse monitoring traps that were used to assess apparent densities of the *G. p. gambiensis* populations in the Niayes^24^ were used to assess the impact of the tsetse control operations on Stomoxinae, Tabanidae and other Diptera.

**Figure 2:**
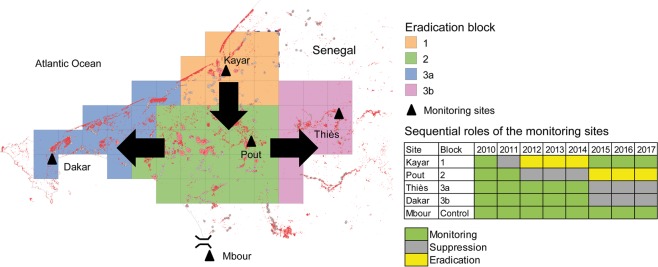
Location of the five sampling locations. The zone with the grids (each cell is 5*5 km) corresponds to the tsetse eradication area. Blocks were tackled sequentially in two phases, i.e. a suppression phase using insecticides and an eradication phase with the sterile insect technique (see text for details). The monitoring of ecological indicators thus followed a stepped wedge approach as shown in the table.

To properly manage the tsetse eradication campaign during the different phases (pre-control monitoring, suppression, eradication and post-eradication monitoring), the entire target area was divided into three operational blocks that were treated sequentially (Fig. 2). During the suppression phase, insecticide-impregnated targets were deployed at an average density of 3.6 per km^2^ and replaced every 6 months. Furthermore, cattle were treated with Vectoclor Plus^TM^ pour-on (Ceva Africa) applied 10 ml for 100 kg of body weight (corresponding to a 5 mg dose of cypermethrin, 7 mg of chlorpyrifos, 5 mg of piperonyl butoxide and 0.5 mg of citronella per kg of body weight) at an average cow density of 2.5 per km²; treatments were irregular and intervals exceeded 6 months. During the eradication phase, 707,040 sterile males were released in block 1 from 2012 to 2015 and 3,643,709 in block 2 from 2015 to 2017. Finally, both the wild and sterile tsetse fly populations were monitored with 24, 72 and 45 biconical traps^24^ in the three blocks respectively. Sampling was done bi-weekly during the eradication phase and monthly during the suppression and monitoring phase. This approach offered a unique opportunity to develop a methodology similar in principle to stepped wedge cluster randomized trials used to assess epidemiological impact of interventions whereby each cluster is assigned to the control treatment initially and clusters are subsequently crossed-over to the intervention in a random selection at fixed time points until eventually all clusters are under treatment^25^.

The main difference is that each cluster was consecutively subjected to two treatment periods, i.e. suppression and eradication, followed by a non-intervention phase of only monitoring (at the time of writing post-eradication monitoring was done only in block 1). In each of five sampling locations (Kayar (block 1), Pout (block 2), Dakar (block 3a), Thies (block 3b) and Mbour centre IRD), the populations of the bioindicators were monitored with 5–6 traps from 2010 to 2017 following the framework presented in Fig. 2. One location was selected outside of the project area as a “control” site where no tsetse control activities were implemented (Mbour centre IRD). The control and environmental monitoring strategies were approved by the Ministère de l’ Environnement of Senegal under the permit 0874/MEPN/DE/DEIE/mbf.

During 114 trap days over the eight years, 14,412 specimens were sampled in the five sampling locations belonging to 31 species including 17 species of Cetoniinae and 9 species of Nymphalidae (see details of species trapped in Table S1). These two families represented 98% of the total specimens sampled indicating good specificity of the monitoring traps. *Pachnoda interrupta*, *Diplognatha gagates* and *Pachnoda marginata* were the most abundant Cetoniinae species sampled (pooled data from all trapping sites) with a mean apparent density of 14.82 ± 46.43, 3.40 ± 8.61 and 1.12 ± 2.28 insects/trap/day, respectively (Fig. S1). These three species were also the most frequent (ratio between positive trapping events and total trapping events) with a frequency of 62.50%, 56.93% and 45.61% for *P. interrupta*, *D. gagates* and *P. marginata*, respectively (Fig. S2). *Charaxes varanes*, *Charaxes epijasius* and *Melanitis leda* were the most abundant species of the Nymphalidae family, with a mean apparent density of 1.04 ± 2.79, 0.76 ± 1.41 and 0.16 ± 0.51 insects/trap/day, respectively (Fig. S1). These three species were also the most frequent with a frequency of 38.34%, 34.80% and 13.68% for *C. varanes*, *C. epijasius* and *M. leda* respectively (Fig. S2).

**Figure 3:**
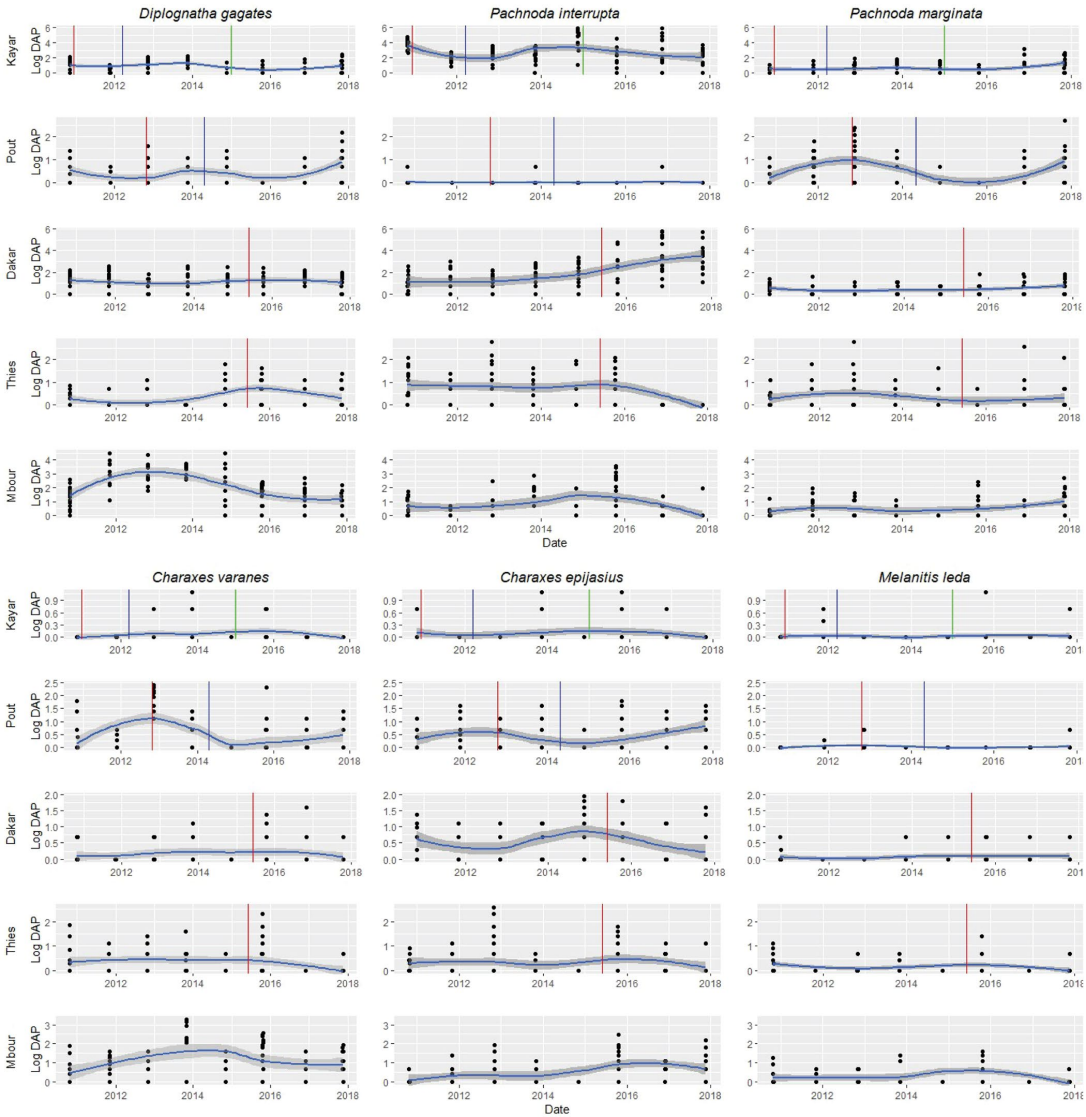
Temporal dynamics of the apparent densities of the most frequent ecological indicators in the eradication area of the Niayes in Senegal (Kayar = Block 1, Pout = Block 2, Dakar and Thiès = block 3, Mbour = control area outside the tsetse-infested zone). The vertical red, blue and green lines correspond to the start of the suppression phase, the start of the eradication phase and the start of the post-eradication monitoring phase, respectively (block 1 only). Cetoniinae species: *Pachnoda interrupta* (Pint), *Diplognatha gagates* (Digag) and *Pachnoda marginata* (Pm); Nymphalidae species: *Charaxes varanes* (Cvar), *Charaxes epijasius* (Cepi) and *Melanitis leda* (Mleda). Blue line is a smooth carried out by Locally Estimated Scatterplot Smoothing Loess method which is a non-parametric regression method adapted point distribution.

Next, we investigated whether the three operational activities (monitoring, suppression and eradication) affected the relative abundance of the six most abundant and frequent ecological indicators (Fig. 3). A linear mixed model was used with the operational activities and time in days elapsed since the last rain (to account for potential climatic variations between years) as fixed effects and the locality and year of survey as random effects (see Methods for details). The analysis indicated no significant impact of any operational activity and seasonality (Tables S2 and S3) on the relative abundance of the bioindicators, with the following exceptions: (i) a significant reduction in the apparent density of *M. leda* in function of time elapsed since the last rain (p = 0.0001, Table S3); (ii) a significant increase in the apparent density of *P. interrupta* during the eradication phase (p = 0.028, Table S2), and (iii) a significant increase in the apparent density of *P. marginata* during the post-eradication monitoring phase (p < 10^−3^, Table S2) and in function of time elapsed since the last rain (p = 0.016).

**Figure 4:**
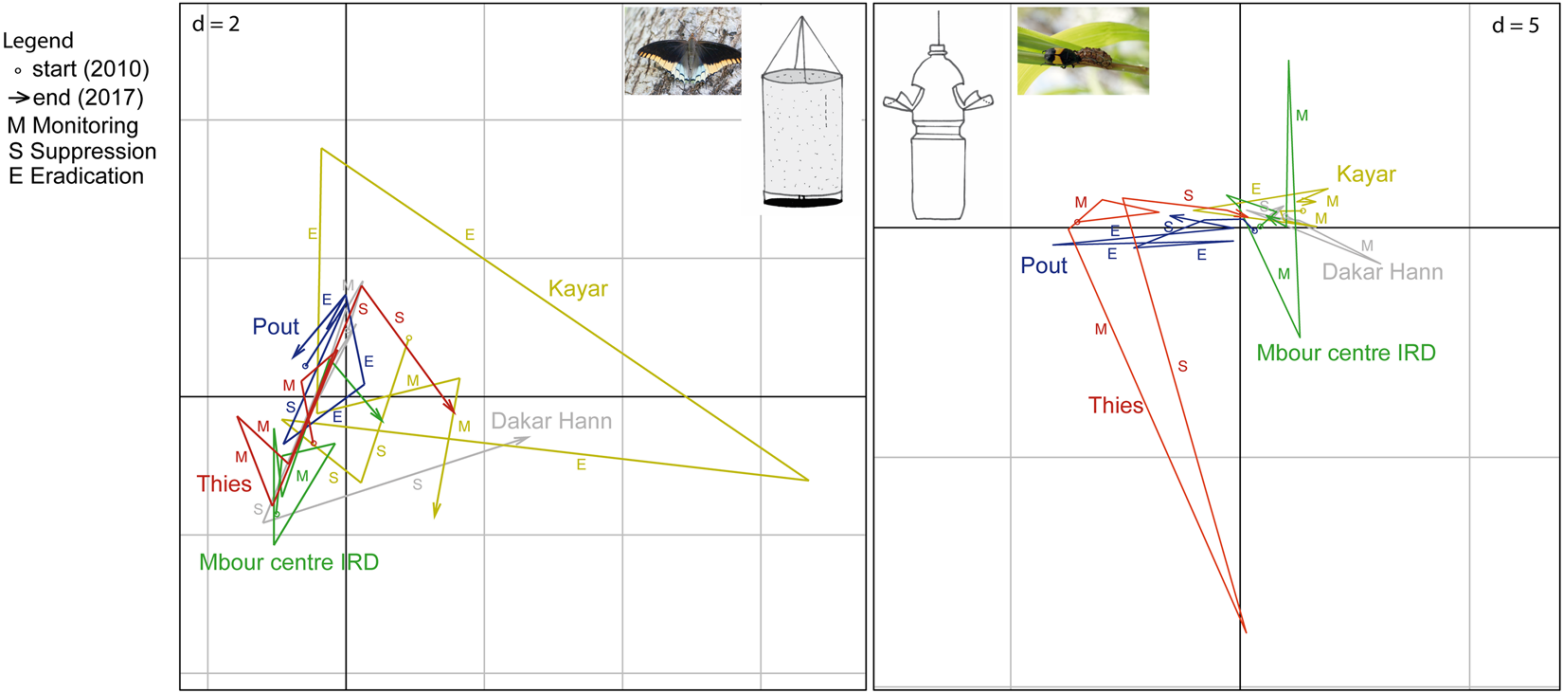
First plan of the triadic analyses of the apparent densities of the most abundant ecological indicators in the study area. The trajectories present the temporal dynamics during eight years in the four sampled locations (Kayar in block 1, Pout in block 2, Thiès and Dakar Hann in block 3 and Mbour centre IRD as a negative control site). The apparent densities of the 3 most abundant species were analyzed and Nymphalidae (left panel) and for Cetoniinae (right panel). Each location is presented with a different color and the letters (M, monitoring, S, suppression and E, eradication) present the three successive phases in each location.

The dynamics of the apparent density of the most abundant ecological indicators were analyzed altogether for each family using a partial triadic analysis (PTA)^26^. This multivariate analysis is used to capture the overall dynamics of an ecosystem based on several species monitored in the same sites at different times. The analysis indicated no homogeneous impact of the suppression and eradication phases on species communities, which would appear as parallel deviations from the initial equilibrium for a given effect (suppression or eradication) in the different locations (Fig. 4). In the different sampling locations of the target area, cyclical evolutions were observed that had similar amplitudes as in the “control area” of Mbour centre IRD, with only two exceptions: (i) a stronger amplitude of the deviation along the second axis in the case of Cetoniinae in Thiès (block 3b) that occurred during a monitoring phase and that was restored during the consecutive suppression phase and (ii) a stronger amplitude of the deviation along the first axis in the case of Nymphalidae in Kayar (block 1) that occurred during the eradication phase and that was restored to pre-control levels during the same eradication phase. Overall, the PTA did not demonstrate any obvious signs of degradation of the ecosystem health as measured by the most abundant ecological indicators altogether.

**Figure 5:**
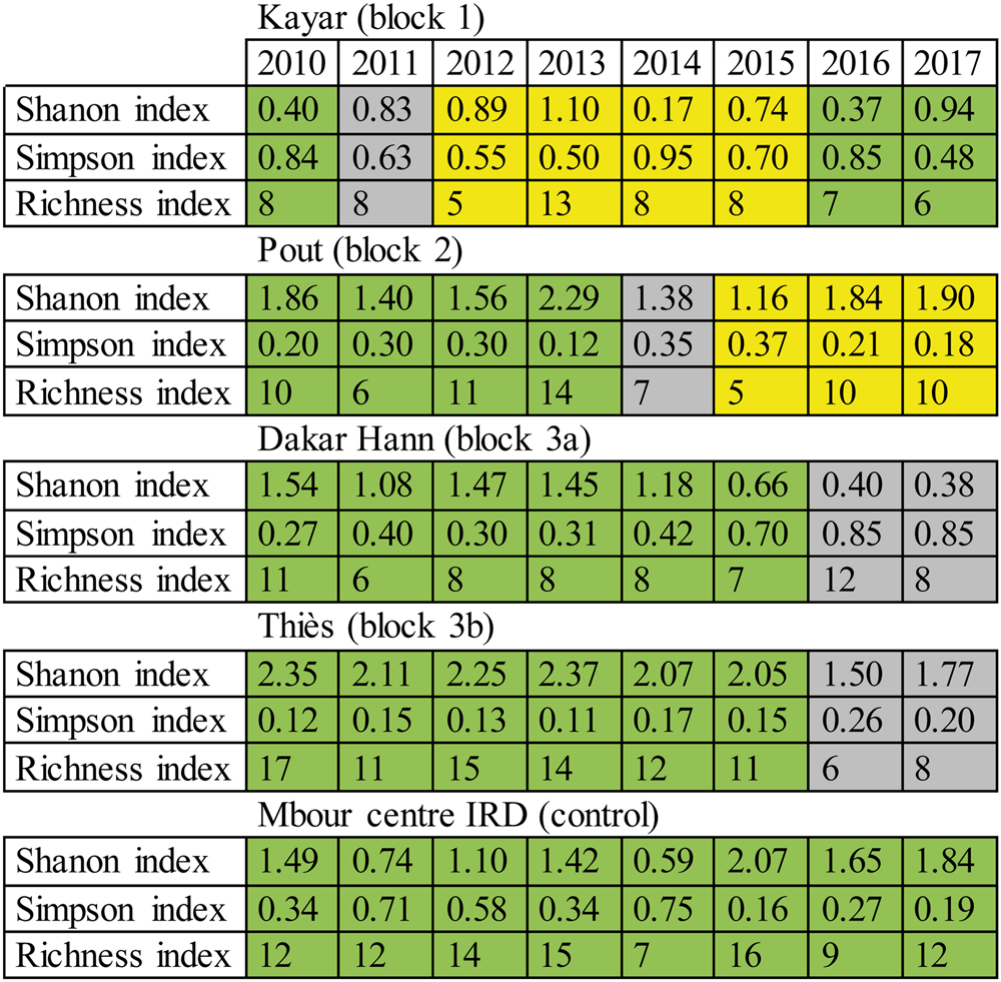
Evolution of three diversity indexes in each location from 2010 to 2017. Green cells present Monitoring periods, grey cells Suppression periods and yellow cells Eradication periods.

Considering the impact of the different control activities on biodiversity indices, species richness was reduced during the insecticide-based suppression phase, but not significantly (p = 0.08 for Cetoniinae and p = 0.09 for Nymphalidae, Fig. 5), and it quickly recovered to pre-suppression levels during the eradication (SIT) phase and during the monitoring phase thereafter. The Shanon index, which increases with ecological diversity, was significantly reduced during the suppression phase for the Cetoniinae (p = 0.03, Fig. 5) but not for the Nymphalidae (p = 0.10). The index recovered to pre-suppression levels during the eradication (SIT) phase and the monitoring phase thereafter (p > 0.75). The Simpson index, which decreases with greater biodiversity, increased marginally (p = 0.06) for Cetoniinae during the suppression phase, followed by a total recovery (p > 0.80) during the eradication and monitoring phases. No significant trend was observed for the Nymphalidae (p > 0.13). It must be noted that all biodiversity indices fluctuated in all locations before the suppression phase as well as in the “control site”, i.e. independently from any of the control activities. These variations might be due to environmental differences (temperature, precipitation) between years.

## Discussion

In this study, two groups of ecological indicators were used that had demonstrated high sensitivity to various human-imposed pressures such as game hunting, transhumance, agricultural activities, etc. in similar ecosystems in Burkina Faso and Niger^21^. For a period of eight years, a large set of sampling data was collected from five locations during the various phases of the tsetse eradication project in the Niayes. The data were subjected to three robust analyses, i.e. the impact on the apparent density of individual species, a partial triadic analysis of the apparent density of the most abundant species, and the temporal dynamics of ecological indices. All analyses indicated a limited but significant negative impact of the suppression phase (insecticide targets and pour-on for cattle) on the Shannon index for Cetoniinae, whereas this effect was marginal for Nymphalidae. Moreover, the Shannon index rapidly recovered to pre-suppression levels during the eradication phase. This small and transient impact of the suppression phase on Cetoniinae was most likely due to the presence of the blue insecticide-impregnated targets, as these insects seem to be attracted to this color as evidenced by their sporadic presence in the tsetse monitoring traps that have the same color and made of the same fabric. However, no evidence of any significant impact was found on other biting flies despite the fact that they were more often exposed to insecticide-treated animals and targets than the studied ecological indicators given that they are hematophagous. The small subset of the Nymphalidae family that can be attracted and trapped with banana traps limits the value of biodiversity indices for this group. However, fruit-feeding species can be sampled in a more standardized way and their apparent densities assessed more accurately in comparison with other species, which have to be trapped with hand nets. The analysis of the dynamics of their apparent densities is thus an accurate proxy of ecosystem health (see the methods section for details).

The biconical traps used to monitor the dynamics of the *G. p. gambiensis* population are also efficient for trapping other Diptera such as Tabanidae and Stomoxinae^27^. Although analyzing the dynamics of the composition of these Diptera was beyond the scope of the present study, an analysis at the family and order level did not show any significant impact of the different operational phases on the apparent density of these flies (p > 0.05, Fig. S3), whereas during the same period, the *G. p. gambiensis* population was eliminated in block 1 and reduced my more than 99% in blocks 2 and 3.

Using pour-on insecticides on cattle may potentially affect litter and soil recycling processes^28^ through their impact on dung beetles but also other Diptera participating in dung recycling^16,17^. In this study, dung beetle communities were not monitored, although Cetonninae larvae have a major role in litter recycling processes. The use of pour-on insecticide in this eradication program was very limited both in terms of frequency and duration, and this is contrary to community-based management of tsetse and trypanosomosis that requires more regular and frequent use for longer periods^2,29,30^.

In this study, we investigated the impact of the eradication project on the environment, caused by the control measures but this was not differentiated from the indirect impact that will result from the intensification of cattle farming in the medium and long term (10–20 years) and that may be beneficial to the environment through a replacement of local cattle with more productive exotic breeds and a better integration between cattle and farming systems^4^. This effect should not be underestimated as overgrazing is one of the major causes of land degradation in Senegal^31^, and cattle emissions gravely contribute to climate change^32^. Therefore, decreasing the number of livestock required per unit product is crucial to decrease greenhouse gas emissions^32^. Moreover, intensification of cattle production systems is expected to lead to a more efficient management of grazing lands and manure^33^ which could substantially decrease emissions as well^32^.

African governments, with the support of the African Union, launched The Pan African Tsetse and Trypanosomosis Eradication Campaign (PATTEC) in 2001 to eradicate tsetse and trypanosomosis continent-wide^34^. Bouyer *et al*. recently discussed ethical considerations on the desirability of the eradication of harmful species^35^ and, considering both intrinsic and instrumental values of tsetse, they suggested that it is ethically defensible to eliminate isolated populations that are main vectors of disease for humans and their domestic animals, such as the *G. p. gambiensis* population targeted in this project^36,37^. The sustained removal of isolated populations of tsetse will only have very limited impact on this tsetse species, which is infesting more than 100,000 km^2^ in Senegal, and much larger areas in West-Africa^36^. Conversely, the successful completion of this project will greatly benefit human and animal health, will significantly increase welfare, will improve the socio-economic status of the farmers, and significantly increase food security in the Niayes area around Dakar where more than 80% of the Senegalese population is living. We recommend that eradication programs should use control tactics that have ecologically the lowest impact and only marginally affect non-target species. Based on the environmental monitoring data presented here, the tsetse eradication program in Senegal is a fine example that tsetse eradication can be achieved with minimal and only temporarily ecological effects.

## Methods

### Use of fruit-feeding insects has ecological indicators

This study focused on fruit-feeding Cetoniinae and Nymphalidae species only because this group was validated as indicating ecosystem health in similar ecosystems in previous studies^21,23^. Both groups were previously evaluated thanks to Brown’s evaluation criteria (1991) and scored 19 each (on a maximum score of 24), and even 23 when evaluated together, like in the present study. The scores considered the following characteristics of these groups:

- both insect families are taxonomically well-known and ecologically highly diversified^38–41^;
- the species selected as indicators are widely distributed in open savannah from the Guinean to the Sahelian regions in Africa and do not display high ecological fidelity;
- the mobility of both insect groups do not affect their indicator value since their densities drop or increase very quickly with distance depending on human activity;
- the selected species are generally caught in high densities and capture scores allow strong statistical analyses, which lead us to switch three of Brown’s criteria (1991) to the maximum level for bait-attracted Nymphalinae in past studies (abundant, non-furtive, easy to find in the field, damped fluctuations, easy to obtain large random samples of species and variation). Seasonal variations, previously described in Burkina Faso^23^, were taken into consideration (see below) and vertical variations are not likely in savannah ecosystems, where the canopy is not continuous and the trees generally small (<10 m).
- the importance of both groups in the ecosystem is probably modest, although the Cetoniinae larvae are saprophytic and play a role in the recycling of organic matter^42^;
- we previously demonstrated that the response of the selected species to ecosystem disturbance is sensitive and analysable^21^. However, the response to disturbance is not linear in the Cetoniinae subfamily, actually favored by a slight disturbance;
- finally, a linear relationship was found between plant species richness and the apparent densities of the most abundant species, which were also correlated together, although belonging to different families. The larval stages of the Nymphalidae species are phytophageous, each species exploiting a restricted number of plant species^43,44^. The larval stages of Cetoniinae eat dead wood and humus^42^, sometime mixed with cow feces and are thus less resource-specific. The relation between plant and insects could thus be related to adult feeding resources, like it was demonstrated before for other butterfly species^45,46^ and beetles^47^.

### Sampling

The trap used for Cetoniinae was a plastic water bottle of 1.5 l, into which two windows of about 8 × 5 cm were made in the upper part. It was free of charge. The trap used for Nymphalidae was constituted by a cylindrical net (60 cm high and 30 cm of diameter) placed 3 cm over a square plank (35 cm sides). It is a classical trap often used in fruit-feeding butterflies survey^48^. In both trapping methods, the bait was decomposed bananas mixed with sugar and exposed to the sun in a closed container for 4 days before being used, as described before^21^.

The use of bait traps only to sample these two families aims at obtaining standardized measure of their density. However, the entities called “Cetoniinae” and “Nymphalidae” in this paper are only subgroups of the family that include the species sampled in this specific way. The bias is stronger for the Nymphalidae family where our sample was mainly composed by the sub-family Charaxinae (Table S1).

Each trap was recorded using a Global Positioning System (GPS) and insects were recorded by species and by trap in order to have the apparent density per trap (ADT). Daily captures in each trap were considered as measurements of the apparent density per trap. In each site, five traps were set during three consecutive days.

### Statistical analyses

For each site, the following indices were calculated: (Sm) species richness, Shannon-Weaver diversity^49^ index (H) and Simpson’s diversity^50^ index (1-D). Sm corresponds to the number of insects per site, H was calculated through this equation $H=-\mathop{\sum }\limits_{i=1}^{n}{p}_{i}\,\log ({p}_{i})$, and D corresponds to $D=\mathop{\sum }\limits_{i=1}^{n}\frac{{n}_{i}({n}_{i}-1)}{N(N-1)}$ where p_i_ corresponds to the proportion species “i” and n_i_ number of species “i”.

Gaussian linear mixed-effects model were used with either the apparent densities of the most frequent ecological indicators or ecological indices (Shanon index, Simpson index and richness index) as response variables, the three treatment regimens (suppression phase, eradication phase and post-eradication monitoring phase) as fixed effect, and year and locations as random effects^51^.

Triadic analysis was used to analyze the global trajectories of the different sites against time, considering the 3 most abundant species for each of the families.

All analyses were performed using R software^52^. nlme package^53^ was used to fit linear mixed-effects models, ade4 package^54^ to fit the PCA and vegan package^55^ to fit the ecological indices.

## Supplementary information

Supplementary Information.

Supplementary Dataset 1.

## Data Availability

All raw data are available as a Supplementary File. All raw data are also deposited in the PREDICT database^56^.
