# Supplementary material for: Environmental impact of tsetse eradication in Senegal
Source: Sci Rep. 2019 Dec 30;9:20313. doi: 10.1038/s41598-019-56919-5 (PMC6937335; doi:10.1038/s41598-019-56919-5)
Supplement: Supplementary file 1 — Supplementary Information. [file 41598_2019_56919_MOESM1_ESM.docx]

**Environmental impact of tsetse eradication in Senegal**

Mamadou Ciss, Mireille D. Bassène, Momar T. Seck, Abdou G. Mbaye, Baba Sall, Assane G. Fall, Marc J.B. Vreysen, Jérémy Bouyer

**Supplementary information**

**Supplementary Tables**

Table S1 List of the species captured. Species of Cetoniinae were diagnosed according to[^1-7^](#_ENREF_1) and with the help of Jean-Philippe Legrand in case of a doubt. Species of Nymphalidae were diagnosed according to[^8^](#_ENREF_8).

| **Genre** | **Species** | **Family / sub-family** | **Code** |
| --- | --- | --- | --- |
| *Charaxes* | *achamenes atlantica* | Nymphalidae | Cach |
| *Charaxes* | *epijasius* | Nymphalidae | Cepi |
| *Polystalactica* | *stellate* | Cetoniinae | Cetnd |
| *Polystalactica* | *punctulata* | Cetoniinae | cetnd2 |
| *Charadronota* | *quadrisignata* | Cetoniinae | Chaqua |
| *Chlorocala* | *guerini* | Cetoniinae | Chlorog |
| *Chondrorrhina* | *abbreviate* | Cetoniinae | Chondab |
| *Charaxes* | *varanes* | Nymphalidae | Cvar |
| *Charaxes* | *viola viola* | Nymphalidae | Cvio |
| *Diplognatha* | *gagates* | Cetoniinae | Digag |
| *Hamanumida* | *daedalus* | Nymphalidae | Hdaed |
| *Hypolimnas* | *misipus* | Nymphalidae | Hypo |
| *Hypolimnas* | *anthedon anthedon* | Nymphalidae | Hypoanth |
| *Melanitis* | *leda* | Nymphalidae | Mleda |
| *Oxythyrea* | *guttifera* | Cetoniinae | Oxygu |
| *Marmylida* | *marginella* | Cetoniinae | Pachno |
| *Pachnoda* | *cordata* | Cetoniinae | Pc |
| *Pachnoda* | *cordata villiersi* | Cetoniinae | Pcvil |
| *Pachnoda* | *interrupta* | Cetoniinae | Pint |
| *Pachnoda* | *marginata* | Cetoniinae | Pm |
| *Polybaphes* | *aequinoctialis* | Cetoniinae | Poaeq |
| *Pachnoda* | *orphanula* | Cetoniinae | Porph |
| *Polybaphes* | *sanguineolenta* | Cetoniinae | Posang |
| *Pseudoprotaetia* | *burmeisteri* | Cetoniinae | Pseudop |
| *Rhabdotis* | *sobrina* | Cetoniinae | Rhsob |
| *Vanessa* | *atalanta* | Nymphalidae | Vanessa |

Table S2 Fixed-effects coefficients of a mixed-effect Gaussian model of the apparent density of three Cetoniinae species (Diplognatha gagates, Pachnoda interrupta and Pachnoda marginata) as response variables, the three treatment regimens (suppression phase, eradication phase and monitoring phase) as fixed effect, and year and locations as random effect.

| *Diplognatha gagates* | | | | | |
| --- | --- | --- | --- | --- | --- |
| Fixed effects | Value | Std. Error | DF | t-value | p-value |
| Intercept | 1.0754 | 0.8142 | 2059 | 1.3208 | 0.1867 |
| Suppression | -0.2972 | 0.6874 | 32 | -0.4323 | 0.6684 |
| Eradication | -0.1783 | 0.5135 | 32 | -0.3472 | 0.7307 |
| Monitoring | -0.2925 | 0.9399 | 32 | -0.3112 | 0.7577 |
| Rainy season | 0.0010 | 0.0181 | 2059 | 0.0536 | 0.9572 |
|  |  |  |  |  |  |
| *Pachnoda interrupta* | | | | | |
| Fixed effects | Value | Std. Error | DF | t-value | p-value |
| Intercept | 0.5738 | 4.8304 | 2059 | 0.1188 | 0.9055 |
| Suppression | 1.1118 | 4.5425 | 32 | 0.2448 | 0.8082 |
| Eradication | 7.6001 | 3.3299 | 32 | 2.3038 | 0.0279 |
| Monitoring | 6.5179 | 5.8350 | 32 | 1.1170 | 0.2723 |
| Rainy season | 0.0358 | 1.1170 | 2059 | 0.3061 | 0.7596 |
|  |  |  |  |  |  |
| *Pachnoda marginata* | | | | | |
| Fixed effects | Value | Std. Error | DF | t-value | p-value |
| Intercept | -0.0312 | 0.1284 | 2059 | -0.2432 | 0.8078 |
| Suppression | -0.0189 | 0.1291 | 32 | -0.1467 | 0.8843 |
| Eradication | 0.0616 | 0.0935 | 32 | 0.6585 | 0.5149 |
| Monitoring | 0.9258 | 0.1594 | 32 | 5.8085 | 0.0000 |
| Rainy season | 0.0083 | 0.0034 | 2059 | 2.4041 | 0.0163 |
|  |  |  |  |  |  |

Table S3 Fixed-effects coefficients of a mixed-effect Gaussian model of the apparent density of three Nymphalidae species (Charaxes varanes, Charaxes epijasius and Melanitis leda) as a response variable, the three treatment regimens (suppression phase, eradication phase and monitoring phase) as fixed effect, and year and locations as random effect.

| *Charaxes varanes* |  |  |  |  |  |
| --- | --- | --- | --- | --- | --- |
| Fixed effects | Value | Std. Error | DF | t-value | p-value |
| Intercept | 0.5888 | 0.2406 | 2059 | 2.4469 | 0.0145 |
| Suppression | -0.1552 | 0.2238 | 32 | -0.6937 | 0.4929 |
| Eradication | -0.1784 | 0.1642 | 32 | -1.0862 | 0.2855 |
| Monitoring | -0.2417 | 0.2914 | 32 | -0.8293 | 0.4131 |
| Rainy season | -0.0069 | 0.0059 | 2059 | -1.1756 | 0.2399 |
|  |  |  |  |  |  |
| *Charaxes epijasius* | | | | | |
| Fixed effects | Value | Std. Error | DF | t-value | p-value |
| Intercept | 0.1948 | 0.0993 | 2059 | 1.9620 | 0.0499 |
| Suppression | -0.0155 | 0.0941 | 32 | -0.1645 | 0.8704 |
| Eradication | -0.0444 | 0.0695 | 32 | -0.6387 | 0.5275 |
| Monitoring | -0.1044 | 0.1224 | 32 | -0.8524 | 0.4003 |
| Rainy season | 0.0009 | 0.0025 | 2059 | 0.3746 | 0.7080 |
|  |  |  |  |  |  |
| *Melanitis leda* |  |  |  |  |  |
| Fixed effects | Value | Std. Error | DF | t-value | p-value |
| Intercept | 0.1390 | 0.0290 | 2059 | 4.7960 | 0.0000 |
| Suppression | 0.0027 | 0.0261 | 32 | 0.1020 | 0.9194 |
| Eradication | -0.0094 | 0.0199 | 32 | -0.4728 | 0.6396 |
| Monitoring | -0.0164 | 0.0363 | 32 | -0.4517 | 0.6545 |
| Rainy season | -0.0028 | 0.0007 | 2059 | -3.8632 | 0.0001 |
|  |  |  |  |  |  |

Table S4 Fixed-effects coefficients of mixed-effect Gaussian models of ecological indices (species richness index, Shanon index and Simpson index) of Cetoniinae as response variables, the three treatment regimens (suppression phase, eradication phase and monitoring phase) as fixed effect, and year and locations as random effect.

| Species richness |  |  |  |  |  |
| --- | --- | --- | --- | --- | --- |
| Fixed effects | Value | Std. Error | DF | t-value | p-value |
| Intercept | 3.8648 | 0.3639 | 128 | 10.6202 | 0.0000 |
| Suppression | -0.9041 | 0.5126 | 25 | -1.7638 | 0.0900 |
| Eradication | -0.2371 | 0.5681 | 25 | -0.4174 | 0.6799 |
| Monitoring | -0.5660 | 0.9056 | 25 | -0.6251 | 0.5376 |
| Shanon index |  |  |  |  |  |
| Fixed effects | Value | Std. Error | DF | t-value | p-value |
| Intercept | 0.8546 | 0.1237 | 128 | 6.9092 | 0.0000 |
| Suppression | -0.3070 | 0.1372 | 25 | -2.2387 | 0.0343 |
| Eradication | -0.0496 | 0.1598 | 25 | -0.3107 | 0.7586 |
| Monitoring | -0.0047 | 0.2519 | 25 | -0.0185 | 0.9854 |
| Simpson index |  |  |  |  |  |
| Fixed effects | Value | Std. Error | DF | t-value | p-value |
| Intercept | 0.5418 | 0.0627 | 128 | 8.6373 | 0.0000 |
| Suppression | 0.1393 | 0.0708 | 25 | 1.9665 | 0.0604 |
| Eradication | 0.0181 | 0.0824 | 25 | 0.2201 | 0.8276 |
| Monitoring | -0.0273 | 0.1299 | 25 | -0.2105 | 0.8350 |

Table S5 Fixed-effects coefficients of mixed-effect Gaussian models of ecological indices (species richness index, Shanon index and Simpson index) of Nymphalidae as response variables, the three treatment regimens (suppression phase, eradication phase and monitoring phase) as fixed effect, and year and locations as random effect.

| Species richness |  |  |  |  |  |
| --- | --- | --- | --- | --- | --- |
| Fixed effects | Value | Std. Error | DF | t-value | p-value |
| Intercept | 2.0663 | 0.1869 | 96 | 11.0561 | 0.0000 |
| Suppression | -0.5484 | 0.3021 | 25 | -1.8154 | 0.0815 |
| Eradication | -0.3231 | 0.3073 | 25 | -1.0514 | 0.3031 |
| Monitoring | -0.5805 | 0.6164 | 25 | -0.9417 | 0.3554 |
|  |  |  |  |  |  |
| Shanon index |  |  |  |  |  |
| Fixed effects | Value | Std. Error | DF | t-value | p-value |
| Intercept | 0.5011 | 0.0652 | 96 | 7.6911 | 0.0000 |
| Suppression | -0.2175 | 0.1303 | 25 | -1.6692 | 0.1076 |
| Eradication | -0.0917 | 0.1228 | 25 | -0.7461 | 0.4626 |
| Monitoring | -0.2314 | 0.2648 | 25 | -0.8739 | 0.3905 |
|  |  |  |  |  |  |
| Simpson index |  |  |  |  |  |
| Fixed effects | Value | Std. Error | DF | t-value | p-value |
| Intercept | 0.6781 | 0.0425 | 117 | 15.9512 | 0.0000 |
| Suppression | 0.1336 | 0.0761 | 117 | 1.7560 | 0.0817 |
| Eradication | 0.08169 | 0.0619 | 117 | 1.3201 | 0.1894 |
| Monitoring | 0.1717 | 0.1544 | 117 | 1.1119 | 0.2685 |

**Supplementary Figures**

Figure S1 Overall mean apparent densities of the monitored species. Top panel: Cetoniinae species, bottom panel: Nymphalidae species. The species codes are presented in Table S1.


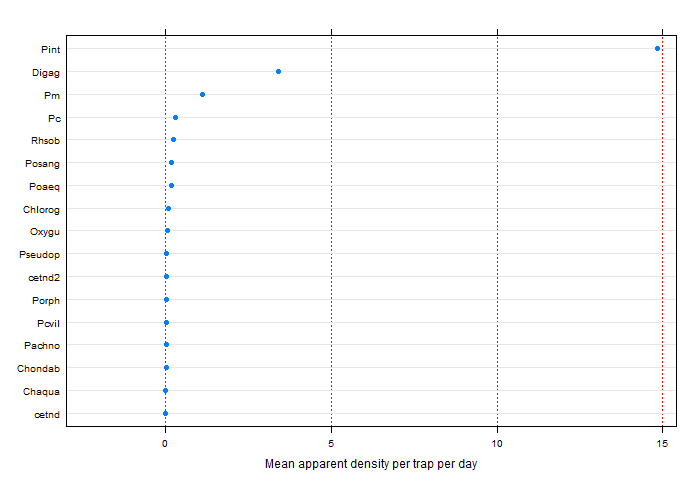


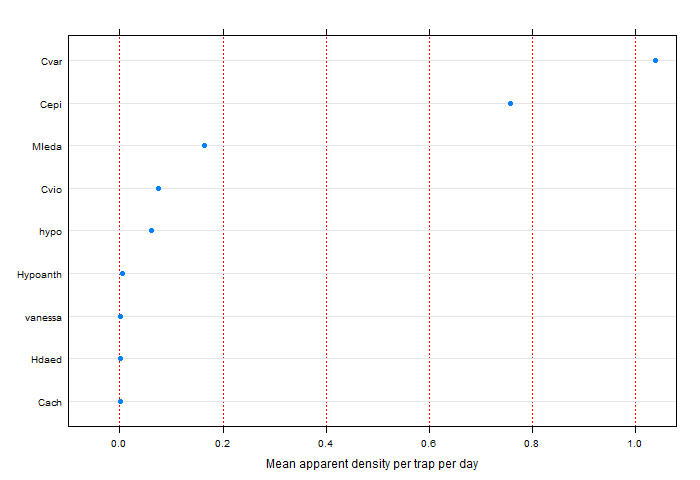


Figure S2 Overall catch frequency of the monitored species. The percentage corresponds to the number of positive trap-days for the species divided by the total trap-days. Top panel: Cetoniinae species, bottom panel: Nymphalidae species. The species codes are presented in Table S1.
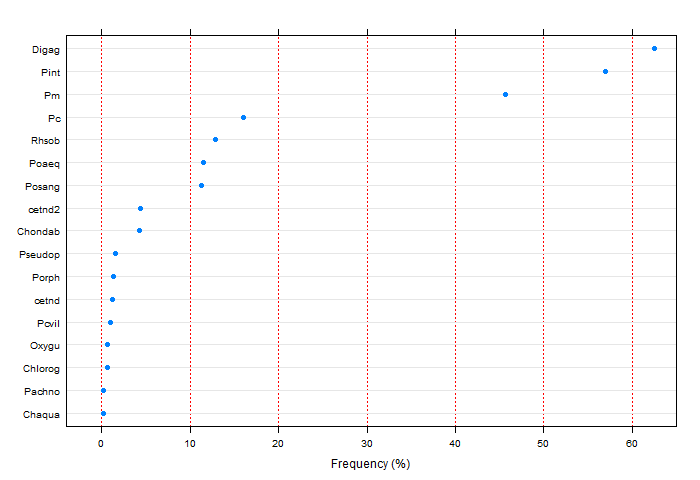

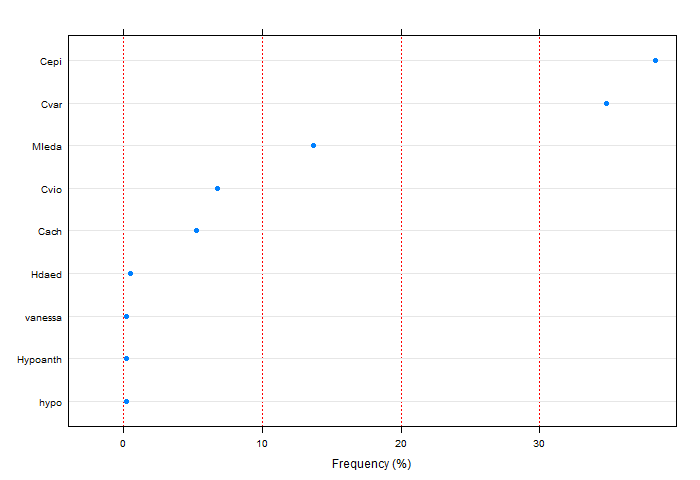


Figure S3 Dynamics of the apparent densities per trap per day of tsetse, Tabanidae, Stomoxes and other Diptera in the different blocks. The vertical red lines correspond to the beginning of the suppression phase, the blue one to the beginning of the eradication phase and the green one to the beginning of the monitoring phase (block 1 only).


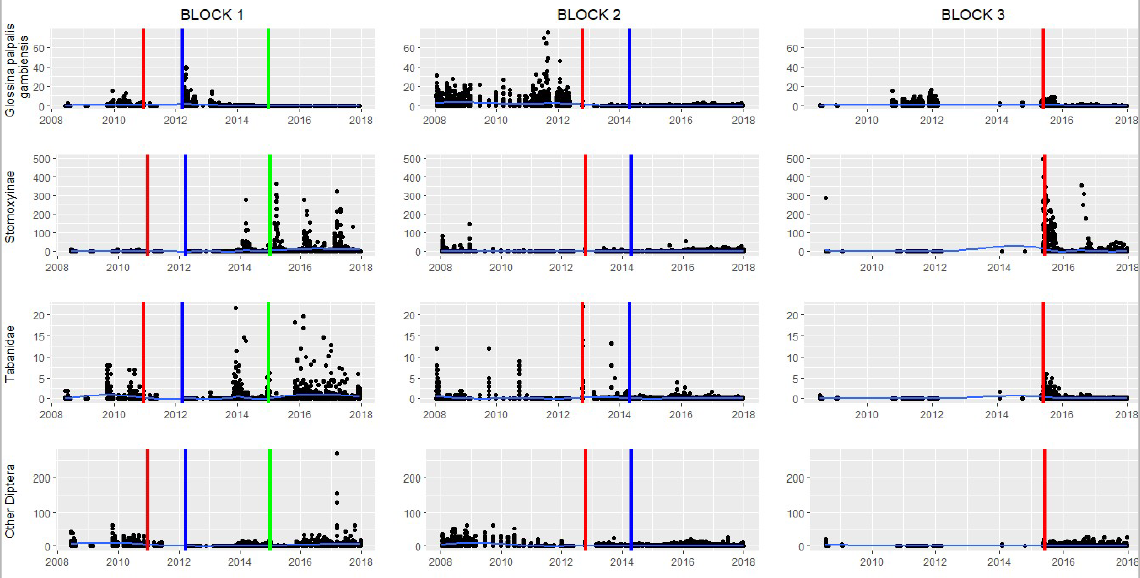


**Supplementary references**

**1 Sakai, K. & Nagai, S. *The Cetoniinae beetles of the world*. (Mushi-Sha, 1998).**

**2 Rigout, J. *Cetoniiini 1*. Vol. 9 (Sciences Nat, 1989).**

**3 Rigout, J. & Allard, V. *Cetoniiini 2*. Vol. 12 (Sciences Nat, 1992).**

**4 Allard, V. *Goliathini 1*. Vol. 3 (Sciences Nat, 1983).**

**5 Allard, V. *Goliathini 2*. Vol. 6 (Sciences Nat, 1985).**

**6 Allard, V. *Goliathini 3*. Vol. 7 (Sciences Nat, 1986).**

**7 Allard, V. *Goliathini 4*. Vol. 11 (Sciences Nat, 1991).**

**8 Larsen, T. B. *Butterflies of West Africa*. (Apollo Books, 2006).**
